# The Reared Male Imago of *Rhoenanthus sapa* Nguyen et Bae, 2004 from China Showing Its Basal Position in the Genus (Ephemeroptera: Potamanthidae)

**DOI:** 10.3390/insects17090894

**Published:** 2026-08-26

**Authors:** Ru-Xuan Hou, Yu-Ning Dong, Chang-Fa Zhou

**Affiliations:** 1 College of Life Sciences, Nanjing Normal University, Nanjing 210023, China; 3836596605@qq.com (R.-X.H.); 2175593241@qq.com (Y.-N.D.); 2 Nanjing Jin Ling High School, Nanjing 210005, China; 3 Nanjing Foreign Language School, Nanjing 210008, China

**Keywords:** imago, mayfly, Oriental realm, taxonomy, evolution

## Abstract

The male imago of *Rhoenanthus sapa* Nguyen et Bae, 2004, which had not been previously reported, was reared for the first time from mature nymph in China. Morphologically, they have lighter bodies and wings and simpler genitalia than congeners. Combined with its nymphal features (relatively short and curved mandibular tusks), this species links the genera *Rhoenanthus* Eaton, 1881 and *Potamanthodes* Ulmer, 1920 together and throws some new light on the evolution of the family Potamanthidae.

## Simple Summary

Previously, the differences between three Asian potamanthid genera (*Rhoenanthus* Eaton, 1881, *Potamanthodes* Ulmer, 1920 and *Potamanthus* Pictet, 1843) were clear. However, the nymphs and female imagos of the species *Rhoenanthus sapa* Nguyen et Bae, 2004 narrowed the gaps between them. Here, the reared male imagos of this species go further; they not only break the distinction between *Rhoenanthus* and *Potamanthodes* but also show a more parsimonious evolutionary pattern with their lightly pigmented wings and *Potamanthodes*-like genitalia. So, their characteristics are described and photographed herein to provide their exact morphology.

## 1. Introduction

The species *Rhoenanthus sapa* was erected upon nymphs and female imagos by Nguyen et Bae, 2004 [1]; its male adult, a key stage for taxonomy, has not been found so far, which impairs its evolutionary position discussion and phylogeny reconstruction.

The genus *Rhoenanthus* Eaton, 1881 is divided into two subgenera comprising nine species [2,3,4], whose nymphs exhibit diverse mandibular tusk morphologies. For instance, in the subgenus *Potamanthindus*, *R. sapa* has dense spine-like tubercles on significantly bent tusks [1,4,5], *R. magnificus* Ulmer, 1920 and *R. obscurus* Navás, 1922 have tubercles at base of tusks whose apical are half narrowed [4,6,7], *R. hunanensis* (You et Gui, 1995) has basal tubercles on almost straight tusks, while *R. youi* (Wu et You, 1986) and *R. coreanus* (Yoon et Bae, 1985) have sparse tubercles on slightly bent tusks [4,8,9,10]. In the other subgenus *Rhoenanthus*, three species (Southeast Asian *R. distafurcus* Bae et McCafferty, 1991, Thai *R. speciosus* Eaton, 1881 and Indian *R. tungaiensis* Balasubramanian et Muthukatturja, 2019) have tooth-like tubercles on elongated tusks [2,3,11,12]. Amongst them, the *R. sapa* has relatively shorter but more bent tusks [1,4,5], which seems more plesiomorphic than others. But this assumption needs its male imago to confirm.

In the original description, Nguyen et Bae (2004) reported that the females of *R. sapa* had unique morphology in the genus *Rhoenanthus*: forewings were stained with light markings or dots but without remarkable stripes or patches, hindwings were almost hyaline and clean, its abdomen had median longitudinal stripes in contrast to the darkly pigmented abdomen of others [1]. This kind of body color pattern is somewhat similar to *Potamanthodes* Ulmer, 1920 or *Potamanthus* Pictet, 1843, which can be tested by its male genitalia [6,13].

Kluge (2004) regarded the relatively long mandibular tusks of *Rhoenanthus* as a plesiomorphy of Potamanthidae, two traditional genera *Rhoenanthus* and *Potamanthodes* which were closer than *Potamanthus* [14]. Differently, Bae et McCafferty (1991) grouped the latter two genera (*Potamanthodes* and *Potamanthus*) together [3]. The nymphs of *R. sapa* have the shortest tusks in the genus *Rhoenanthus*, which seems more apomorphic than others under Kluge’s hypothesis or assumption of Bae et McCafferty, so their phylogenetic ideas can also be tested by their male morphology.

McCafferty et Bae (1992) and Mayorga et al. (2024) observed some behaviors of potamanthid mayflies, including aggressive fighting of *Rhoenanthus coreanus* and filtering of *Anthopotamus verticis* (Say, 1839) [13,14,15,16,17]. More observation on rearing potamanthids will show the exact function of their tusks and elongated forelegs.

In spring 2026, we had a collection trip in Yunnan province, southwestern China. During this collection, more than thirty *R. sapa* nymphs were sampled, and around 25 mature ones were moved into our lab to rear into adults. After about two weeks, most nymphs became adults. Those materials provided us an opportunity to demonstrate the male of *R. sapa*, to know some biology of its nymphs and to discuss its phylogeny.

## 2. Materials and Methods

### 2.1. Materials

China: 5 nymphs, Xili River, Tuanjie town, Yunlong County, leg. by Hui Xie, Yan-Yan Jia, Ping Chen, VII-2008; 22 nymphs, Gu River, Jingdong County, 15-IV-2001, leg. by Chang-Fa Zhou; 10 nymphs, Fengshan town, Jinggu County, leg. by Chang-Fa Zhou, 7-IV-2001; 13 nymphs, 5 male imagos, 6 female imagos, 2 male subimagos, 2 female subimagos (adults reared from nymphs), Tong-Bi-Guan village, Yinjiang County, Yunnan Province, 24.6410 °N, 97.6619 °E, alt. 1358 m, 17-IV-2026, leg. by De-Wen Gong and Xu-Hong-Yi Zheng.

### 2.2. Specimen Collection and Photography Documentation

Nymphs were collected by hand nets, and adults were reared from mature nymphs. All materials were stored in ethanol (≥80%) and deposited in the Mayfly Collection, College of Life Sciences, Nanjing Normal University (NNU).

During rearing, the mature nymphs were gathered into a tray with dechlorinated water (tap water under sunlight for a couple of hours in open bucket), small stones, twigs and leaves. Then, the whole system was covered by a small tent made of mosquito net. During the whole rearing process, a small inflator was employed to add air to water. After subimagos appeared, most of them were undisturbed to obtain imagos. The dead nymphs, several subimagos and all imagos along with their nymphal exuviae were collected for further study.

The morphological characters were documented through multiple imaging techniques. Whole-body images of nymphs and adults were obtained using a Canon EOS 90D camera (Canon Company, Suzhou, China). For intermediate-sized structures like male genitalia and hindwing, a stereomicroscope (Nikon SMZ1000, Nikon Company, Shanghai, China) with a Mshot Image Analysis System (Mshot MZ81, Guangzhou, China) were used. Eggs were visualized using a Thermo Fisher Scientific Apreo 2S scanning electron microscope (Thermo Fisher Scientific Company, Waltham, MA, USA).

Eggs for scanning electron microscopy (SEM) were dissected from the female imago and prepared according to a standard protocol: dehydrated through an acetone gradient (70%, 80%, 90%, 98%, 100%, 10–20 min each), followed by treatment with 1–1.5 mL hexamethyldisilazane (HMDS) for 30 min. The samples were then left in a vacuum container for desiccation overnight before being sputter-coated with a gold film prior to imaging.

### 2.3. Molecular Comparison

DNA samples were collected from the thoracic muscles of one female imago to sequence its mitochondrial *COI* gene. The PCR amplification adopted Premix Taq (Takara Bio Inc., Beijing, China) with forward primer LCO (5′-TTC AGC CAC TTT ACC GCG-3′, and reverse primer HCO2198 (5′-TAA ACT TCA GGG TGA CCA AAA AAT CA-3′. PCR conditions were initial denaturation at 94 °C for 5 min, 40 cycles of denaturation at 94 °C for 30 s, annealing at 50 °C for 30 s and extension at 72 °C for 40 s, with final extension at 72 °C for 10 min. After that, the *COI* sequence data of this species was submitted to GenBank. A total of five related sequences in same genus were downloaded from the GenBank for further comparison and analysis. Five sequences were aligned via the software MEGA 11. The final sequences include 658 bp after deleting the ambiguous positions, and their GenBank accession numbers are listed in Table 1.

The genetic distances of those six sequences were analyzed and performed by using the Kimura 2-parameter model in the MEGA 11 software.

## 3. Results

### 3.1. Descriptions

*Rhoenanthus sapa* Nguyen et Bae, 2004

*Rhoenanthus* (*Potamanthindus*) *sapa* Nguyen et Bae, 2004: 13. Types: nymph, female, from Vietnam [1].

*Rhoenanthus* (*Potamanthindus*) *sapa*: Nguyen et Bae, 2006: 23 (biology, key); Han, Zhang et Zhou, 2021: 573 (nymphal photo, first record from China); Vasanth et al., 2024: 273 (nymph, first record from India, possible misidentification, see discussion below) [4,5,18].

**Figure 1:**
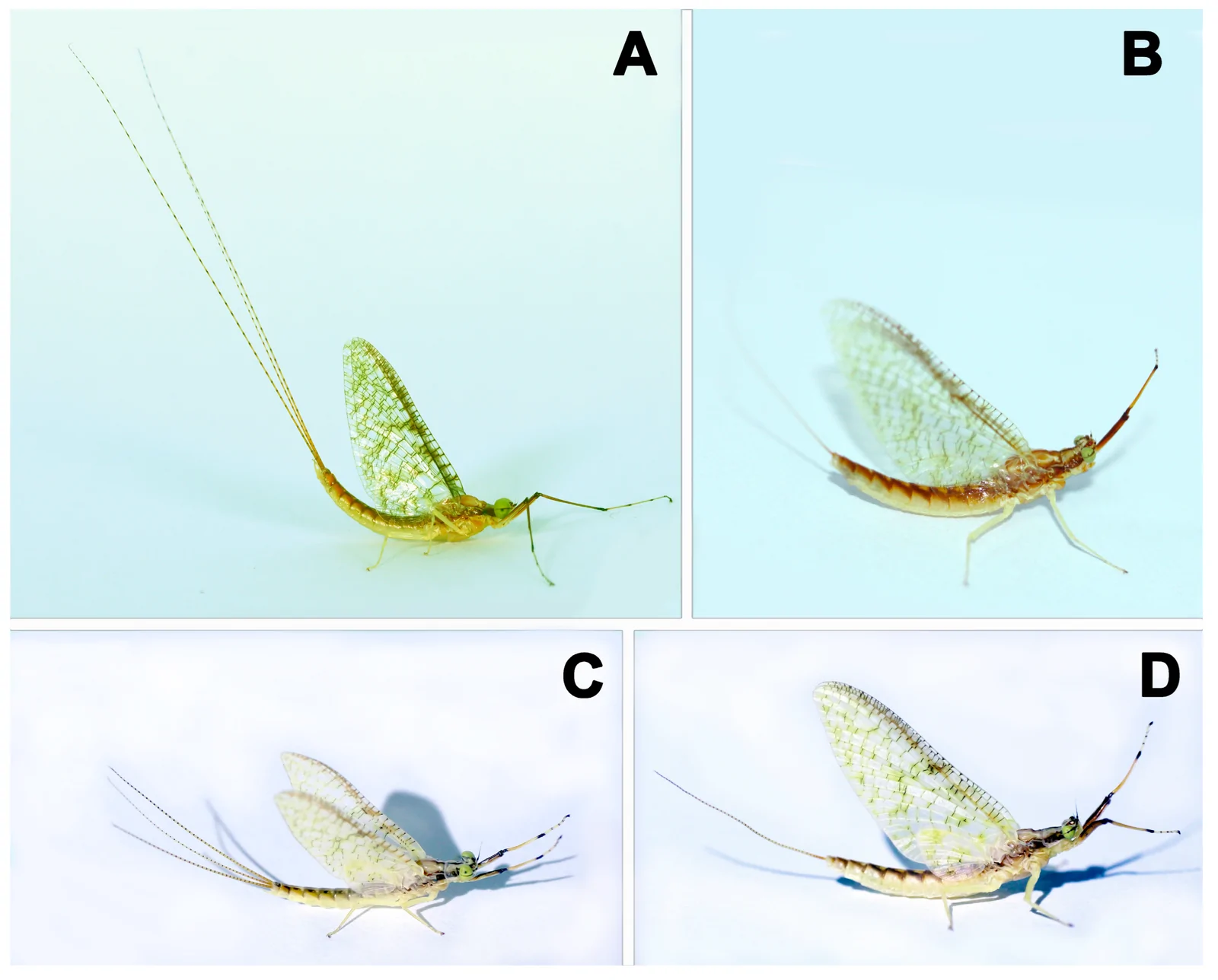
Living habitus of *Rhoenanthus sapa* adults. (**A**). Male imago; (**B**). female imago; (**C**). male subimago; and (**D**). female subimago.

**Figure 2:**
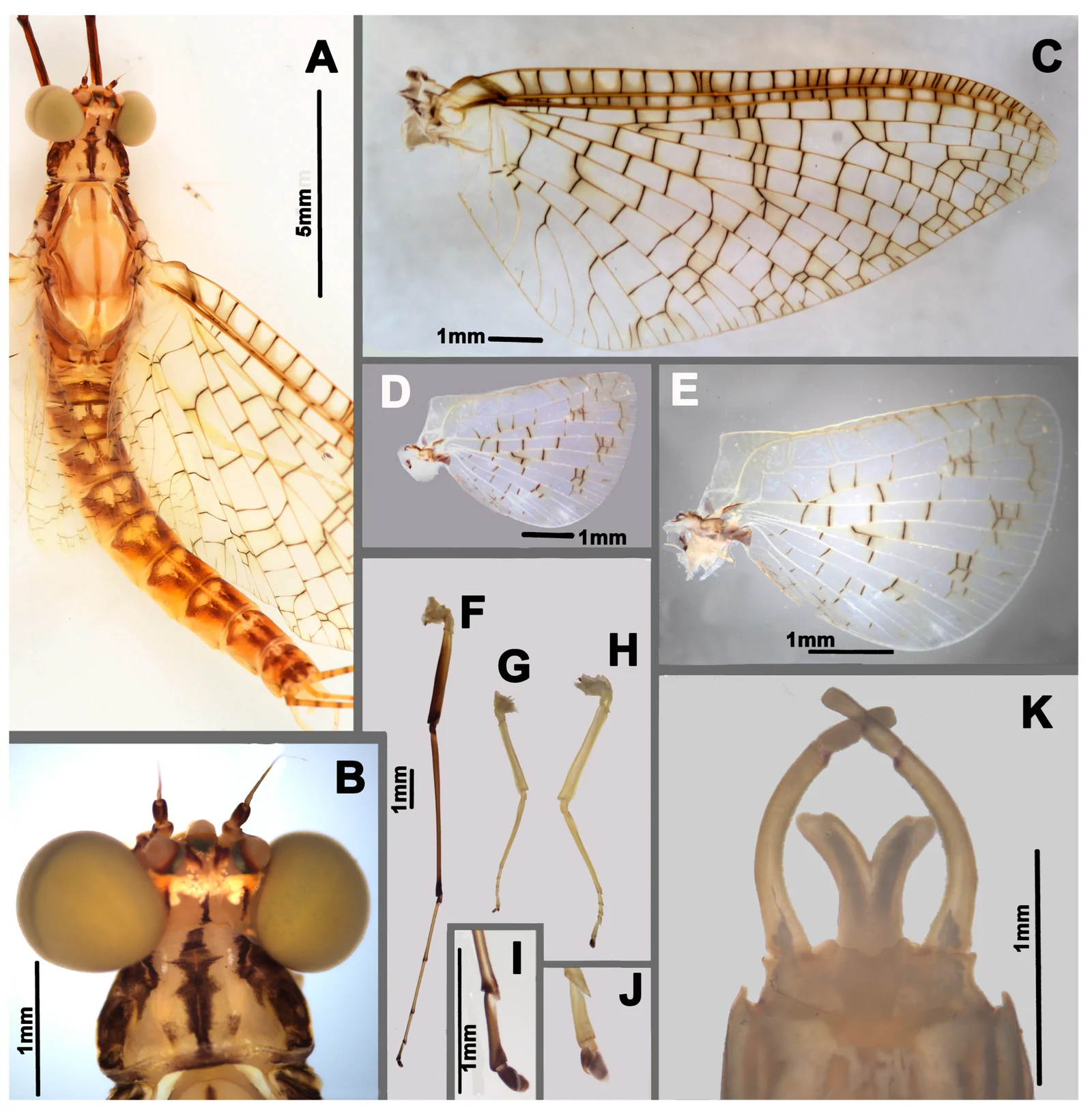
Characters of male imago of *Rhoenanthus sapa*. (**A**). Body (dorsal view); (**B**). head (dorsal view); (**C**). forewing; (**D**). hindwing; (**E**). hindwing enlarged; (**F**). foreleg; (**G**). midleg; (**H**). hindleg; (**I**). foreclaw; (**J**). midclaw; and (**K**). genitalia (ventral view).

Male imago (in alcohol, Figure 1A and Figure 2): body length 12.0–15.0 mm, forelegs 10.5–12.0 mm, forewings 11.0–12.0 mm, hindwings 3.5–4.5 mm, terminal filament 20–21.5 mm, cerci 25.0–31.0 mm. Body pale yellowish, with yellowish brown stripes and markings on wings and body (Figure 1A and Figure 2A). Antennae pale yellow but scape with reddish black apical ring, pedicel brown (Figures 2A,B). Ocelli on anterior margin of head and between compound eyes, with brown base and pale apex (Figures 2A,B). Compound eyes on lateral sides of head, with gray basal half and greenish apical half (Figures 2A,B); shortest distance between two eyes about 0.7× diameter of one eye in dorsal view (Figure 2B). Vertex with three longitudinal stripes, one at midline, other two beside eyes (Figure 2B). Thoracic nota with reddish midline and lateral margins; those stripes on pronotum more distinct than others (Figure 2A). Forewings transparent but longitudinal veins yellowish brown, all crossveins pigmented deep brown and washed with surrounding yellowish cloud, particularly those of costal and subcostal sections; those two sections with more crossveins than other parts (Figure 2C). Bullae of Sc, Rs_1_, Rs_5_ and MP_1_ distinct; MP_2_ connecting CuA at base with two short crossveins (Figure 2C). Hindwings paler than forewings but with pigmented crossveins and clouds (Figures 2D,E); costal projection distinct, R_1_ curved towards Sc at base clearly, some zigzagged; MA single, MP close to CuA at base, MP_2_ indistinct (Figure 2E). Forelegs deep brown, tarsi slightly paler; length ratio of forefemora:tibiae:tarsi = 1.0:1.8:1.8; length ratio of five segments of tarsi from base to apex = 1.0:8.0:7.6:4.0:2.8 (Figure 2F). Two claws of foreleg brown, similar, blunt (Figure 2I). Mid- and hind-legs pale yellowish, only claw brown, two claws different, one acute, one blunt (Figure 2J). Mid- and hind- tibiae with patellar-tibial fusion suture. Length ratio of midfemora:tibiae:tarsi = 1.0:0.9:0.6; length ratio of four segments of tarsi from base to apex = 1.0:0.8:0.5:1.3 (Figure 2G); length ratio of hind femora:tibiae:tarsi = 1.0:0.9:0.5; length ratio of four segments of tarsi from base to apex = 1.0:0.8:0.8:1.2 (Figure 2H).

Abdomen with reddish terga but each of them with a pale midline and a pair of submedian dots; those three pale parts usually merged together forming regular markings (Figure 2A). Sterna pale yellowish. Genitalia pale yellowish; forceps with brown articulations (Figure 2K); subgenital plate with wide V-shaped concave posterior margin; length ratio of three segments of forceps = 1.0:0.2:0.2, segment I smoothly curved inwards; penes reaching 2/3 length of segment I of forceps, basal 2/3 fused together while apical 1/3 diverged laterally, leaving a clear V-shaped cleft between two penes; apex of penis blunt, with a very shallow notch (Figure 2K). Terminal filaments subequal to cerci in thickness but slightly shorter in length, articulations of caudal filaments yellow to brown, other parts pale yellowish (Figure 1A).

**Figure 3:**
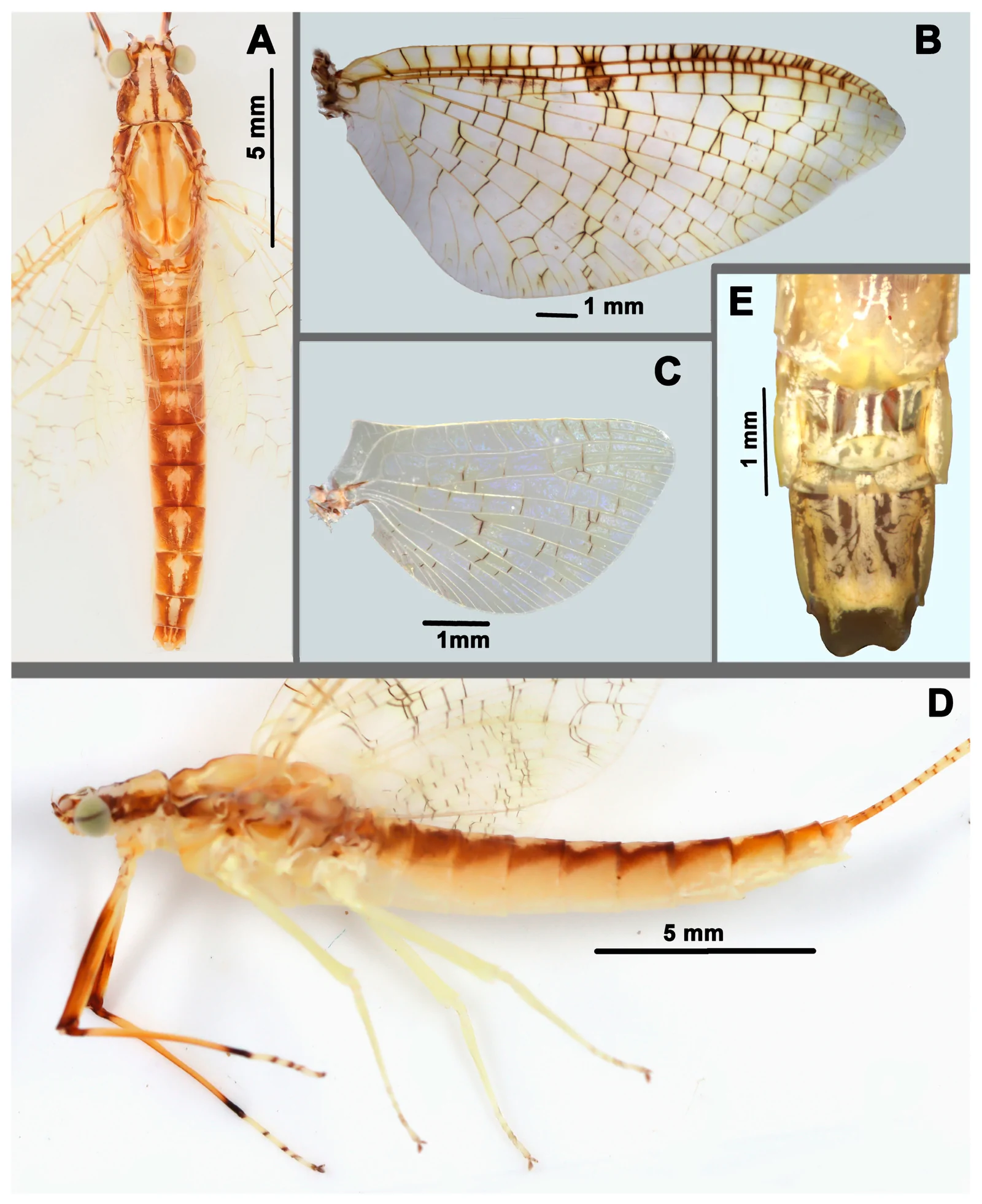
Characters of female imago of *Rhoenanthus sapa*. (**A**). Body (dorsal view); (**B**). forewing; (**C**). hindwing; (**D**). body (lateral view); (**E**). terminal abdomen (ventral view).

Female imago (in alcohol, Figure 1B and Figure 3): descriptive data complement to Nguyen et Bae (2004) [1]: body length 16.0–19.0 mm, forelegs 8.0–10.5 mm, forewings 15.0–18.0 mm, hindwings 5.5–6.0 mm, terminal filament 16.0–20.0 mm, cerci 19.0–25.0 mm. Similar to male in body pattern and color but paler, in particularly the legs and wings (Figure 1B). Shorter distance between two compound eyes about 1.8× diameter of one eye in dorsal view (Figure 3A). Stripes on head and thorax slightly wider and deeper than male imago (Figures 3A,D). Forewings slightly paler than male (Figure 3B). MP_2_ of hindwing more distinct and longer than that of male (Figure 3C). Length ratio of forefemora:tibiae:tarsi = 1.0:0.9:0.6; length ratio of five segments of foretarsi from base to apex = 1.0:2.5:2.0:1.0:2.5 (Figure 3D); ratio of midfemora:tibiae:tarsi = 1.0:1.0:0.5; length ratio of four segments of midtarsi from base to apex = 1.0:0.7:0.5:1.0; length ratio of hindfemora:tibiae:tarsi = 1.0:0.8:0.4; length ratio of four segments of hindtarsi from base to apex = 1.0:0.8:0.5:1.3; two claws of all legs similar, one blunt, one acute (Figure 3D). Posterior margin of sternum VII convex, sternum IX with smoothly concave posterior margin (Figure 3E).

Male subimago (in alcohol, Figure 1C): body 16.0 mm, forewings 14.0 mm, hindwings 5.0 mm, forelegs 8.0 mm, caudal filaments 16.5 mm; length ratio of forefemora:tibiae:tarsi = 1.0:1.2:1.0. Similar to male imago in color but duller, especially wings.

Female subimago (in alcohol, Figure 1D): body 16.0 mm, forewings 16.0 mm, hindwings 5.0 mm, forelegs 6.0 mm, caudal filaments 13.0 mm, length ratio of forefemora:tibiae:tarsi = 1.0:1.1:0.6. Similar to female imago in color but duller, especially wings.

**Figure 4:**
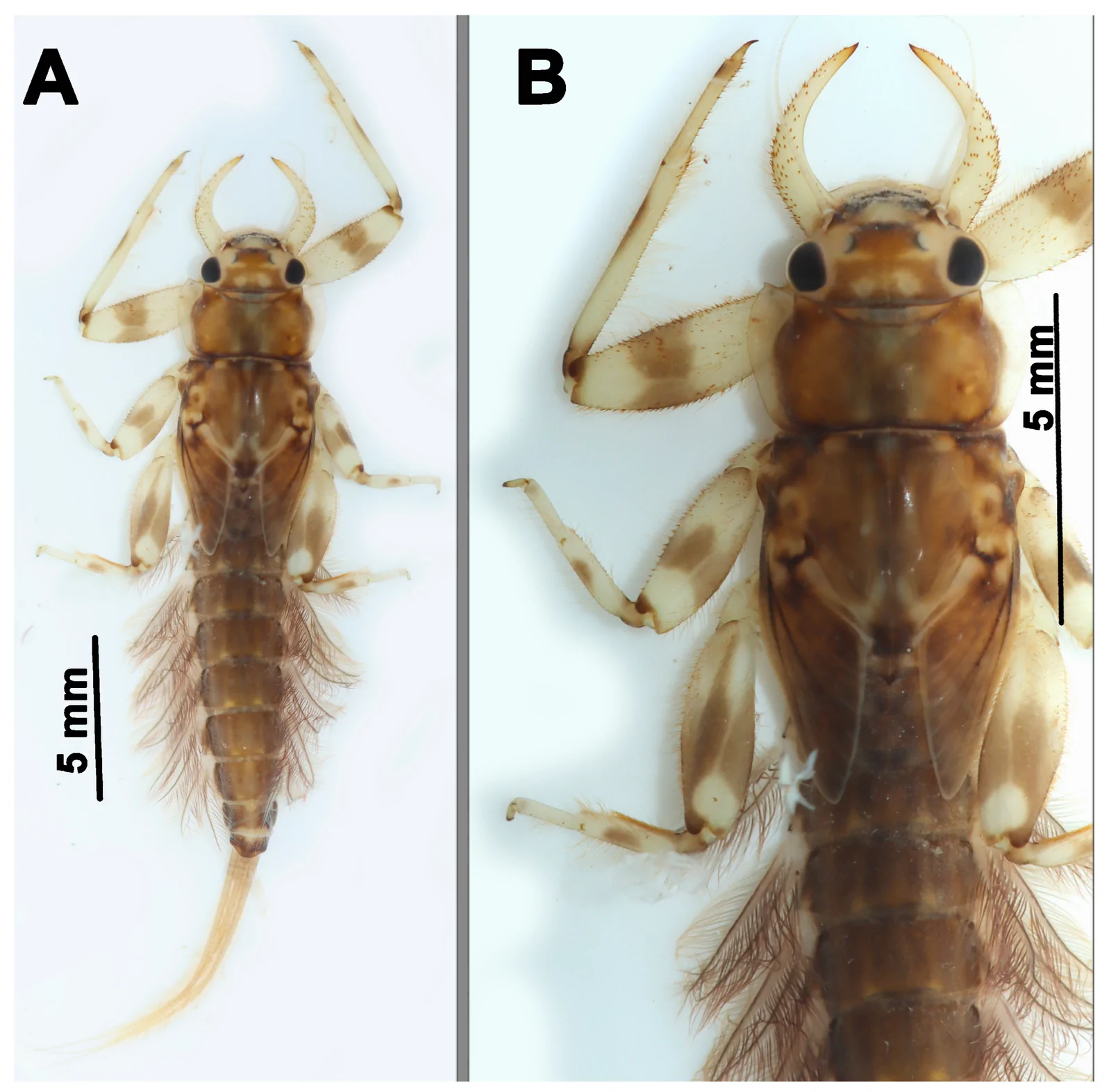
Nymphal habitus of *Rhoenanthus sapa*. (**A**). Body (dorsal view); (**B**). head and thorax (dorsal view, showing the mandibular tusks and forelegs).

Mature nymph (in alcohol): descriptive data complementary to Nguyen et Bae (2004) [1]: body length 17.0–21.0 mm, caudal filament 9.0–10.0 mm, mandibular tusks 3.0–3.5 mm. Body yellowish brown to brown (Figure 4). Mandibular tusks strongly curved inwards, surface with tiny tubercles and hair-like setae; dorsal surface of femora, tibiae and tarsi of forelegs with hair-like setae and spine-like setae, those of margins longer and denser than others; length ratio of those segments = 1.0:1.2:0.7 (Figure 4).

**Figure 5:**
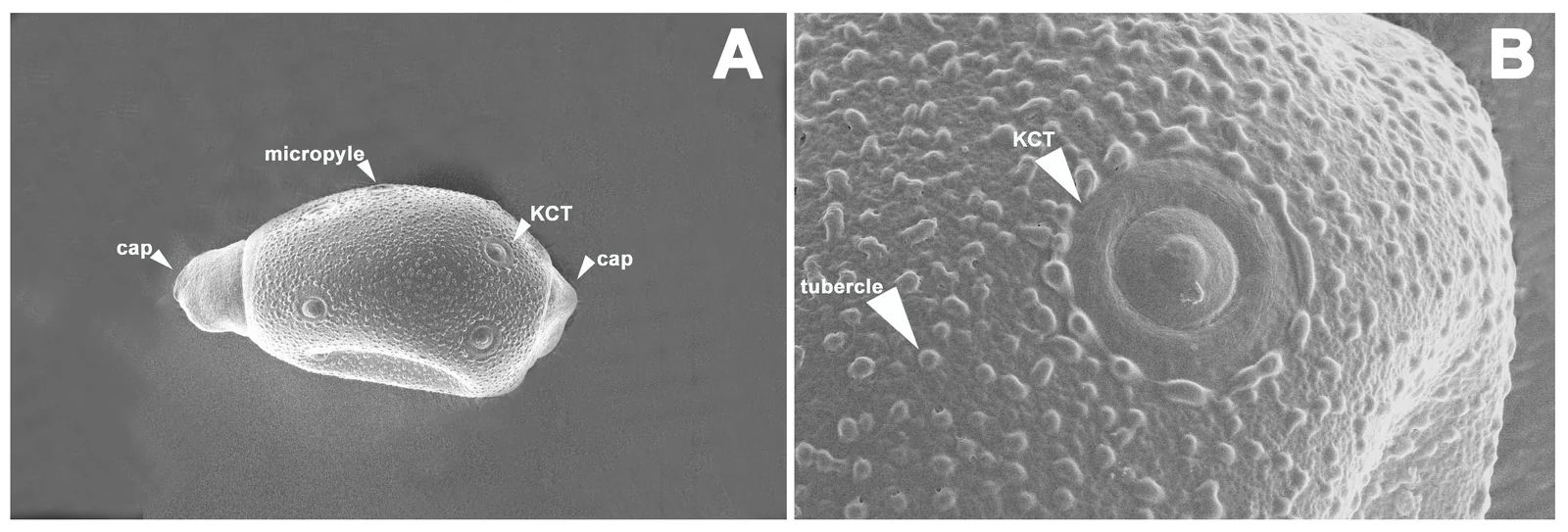
SEM picture of *Rhoenanthus sapa* egg. (**A**). Whole egg; (**B**). KCT and tubercle-like structures enlarged.

Egg (Figure 5): roughly olive-shaped, both poles with cap; two circles of distinct KCTs (knob-terminated coiled threads) near poles or caps (Figures 5A,B); micropyle oval, near equator of egg (Figure 5A). Whole surface decorated with dense tubercle-like structures (Figure 5B).

### 3.2. Feeding Behavior of Nymphs

Four types of feeding behavior of *R. sapa* nymphs were observed and recorded on cellphone videos: (1) direct scraping: nymphs scrape algae or debris on stone surface with their mouthparts. (2) direct collecting: nymphs collect algae or debris on stone surface or broken leaves in water using their foreclaws and then deliver food directly to mouthparts. (3) cooperative scraping: nymphs use their forelegs to grasp a small stone or pebble, move it under its head, holding it with forelegs and mandibular tusks, and let mouthparts scrape its surface for food. Nymphs can change or roll the pebble for more food during the scraping. (4) feeding on forelegs: nymphs stretch out their forelegs and swing them inwards, collect or filter the debris in water, then move their forelegs under mouthparts and scrape food on legs.

The nymphs usually live in interstitial microhabitats. The burrowing of them was not observed.

### 3.3. Habitat

**Figure 6:**
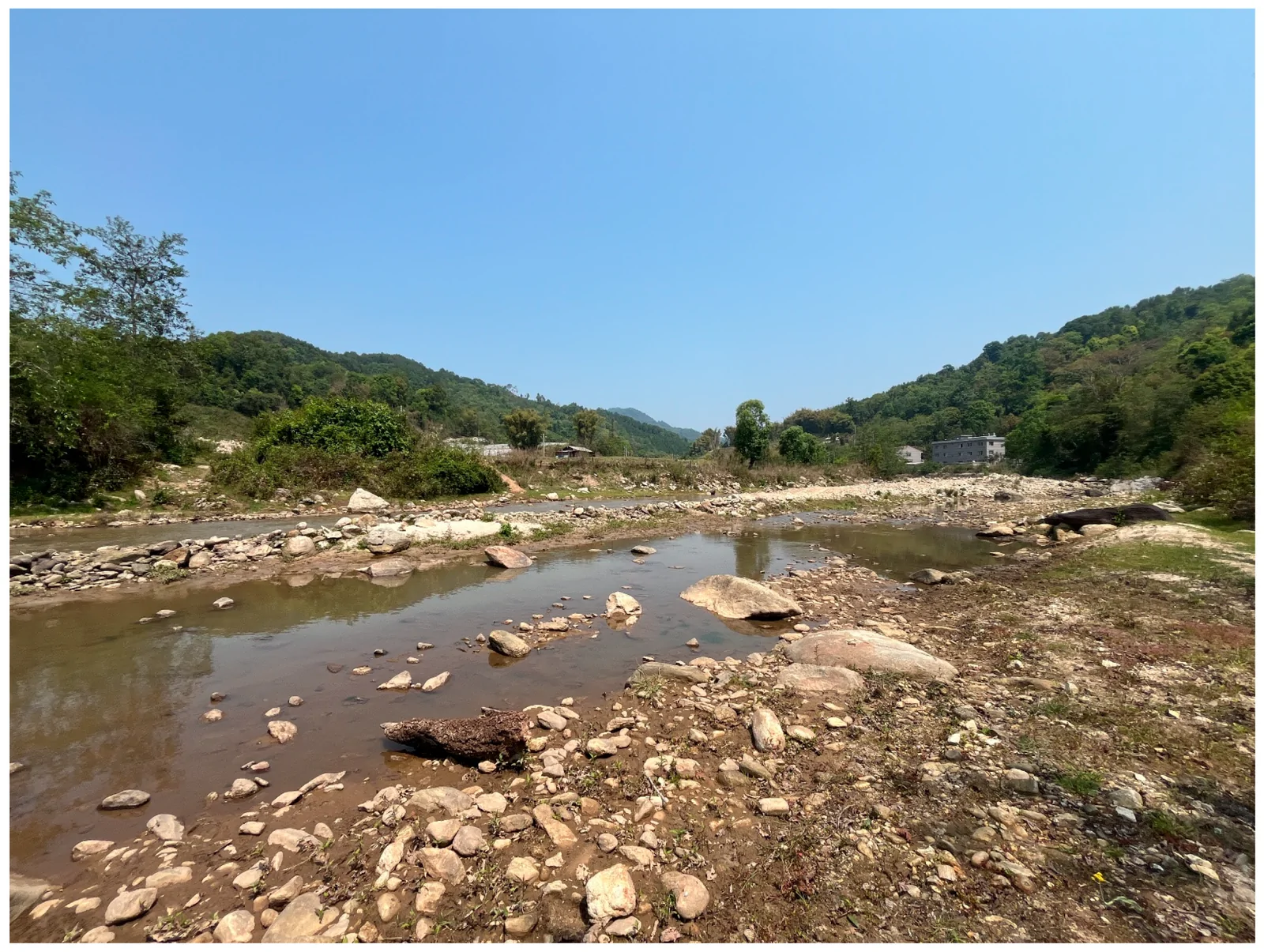
Typical habitat of *Rhoenanthus sapa* nymphs (Yunnan province, China).

Nymphs of *R. sapa* in this study were collected from several locations; the widths of their living streams or rivers are from 20 m to 50 m, and most of them were collected in lentic sections of water, where velocity is less than 0.5 m/s. Bottoms of water were composed of different-sized rocks, gravels, peddles and sand, and mud (Figure 6).

### 3.4. Diagnosis and Comparison

First, unlike all other congeners with distinctly reddish-brown markings on wings, both male and female imagos of *Rhoenanthus sapa* have yellowish brown clouds or light markings on wings (Figure 1). Second, the abdomen of this species has median longitudinal stripes while others usually have lateral stripes or a totally brown body (Figure 1, Figure 2A and Figure 3A,D). Third, this species (less than 20 mm) is usually smaller than others (more than 20 mm). Fourth, the male genitalia is smaller and its penes are thinner than others (Figure 2K). The penes of *R. coreanus*, *R. magnificus* and *R. obscurus* are much wider; forceps and penes of *R. hunanensis* and *R. youi* are distinctly longer and straighter [3,4,5]. In sum, this species can be identified by its light color and short genitalia.

In the subgenus *Potamanthindus* of *Rhoenanthus*, *R. magnificus*, *R. obscurus* and *R. coreanus* have stout penes; the other two *R. hunanensis* and *R. youi* have slim and forked penes, but their penes are much longer than *R. sapa*, and the penial apex of them are tapered, in contrast to the blunt penial apex of *R. sapa*. All the above four species have conspicuous reddish-brown stripes on body and wings [3,4].

The nymphs of *R. sapa* have strongly incurved mandibular tusks, their dorsal surface covered with dense spines or tubercles (Figure 4). The other species in the same subgenus either have spines on the widened base of tusks (*R. magnificus*, *R. obscurus*), or smoothly curved tusks with basal spines (*R. hunanensis*), or have very sparse spines (*R. youi* and *R. coreanus*) [3,4,5].

The eggs of *R. sapa* are very close to other *Rhoenanthus* species, like *R. hunanensis* [4] or *R. speciosus* [11]. All of them have two caps, two circles of KCTs and one equatorial micropyle. But different to some *Potamanthodes* species [19], the KCTs of *Rhoenanthus* seem further to poles than *Potamanthodes*.

### 3.5. Molecular Distance

The K2P values of *Rhoenanthus sapa* to other two congeners are more than 23%, such as 23.5–26.4% to Korean *R. coreanus*, 24.7–24.8% to an unnamed *Rhoenanthus* species from Thailand. These data show that *R. sapa* is an independent species (Table 2).

## 4. Discussion

The notable nymphal mandibular tusks (Figure 4) and the asymmetrically forked MP in hindwings (Figure 2D,E and Figure 3C) of *R. sapa* show that it is a member of the genus *Rhoenanthus*. However, it has *Potamanthodes*-type body color and male genitalia (Figure 1 and Figure 2C–E,K). Its male genitalia is extremely similar to some *Potamanthodes* species, such as *P. macrophthalmus* You, 1984 or *P. curvativus* Qu et al., 2026 in general shape and blunt apex of penes [19,20]; its hindwing venation is close to that of *P. yooni* (Bae et McCafferty, 1991) in curved R_1_ [3,19]. Furthermore, some *Potamanthodes* species, like *P. idiocerus* (Bae et McCafferty, 1991), have relatively distinct mandibular tusks [3]. So, the male imago of *R. sapa* described here support Kluge’s opinion that the two taxa (*Rhoenanthus*, *Potamanthodes*) are closer than the third one *Potamanthus*.

Bae et McCaffery (1991, 1993) and Kluge (2004) suggested that the shortened mandibular tusks are the evolutionary trend in the clade *Rhoenanthus*+*Potamanthodes* or Potamanthidae [3,14,21]. Following this suggestion, the *R. sapa* is more derived than other *Rhoenanthus* species but closer to *Potamanthodes*; this point is also supported by its *Potamanthodes*-like venation, color, male genitalia and subequal length of caudal filaments.

But a converse scenario is also possible because generally, the tusks may have evolved from small to large, nymphal forelegs from short to long, body from small to big, and imaginal MP of hindwing forked from symmetrically to asymmetrically, penes from simple to complex. So we hypothesized here that the common ancestor of Potamanthidae is *Potamanthus*-like, nymphs without mandibular tusks and subapical spinous rows on forefemora, straight running R_1_ and symmetrically forked MP of hindwings, imaginal wings with distinct markings and simple tubular penes. Later, this primitive type evolved into three clades. *Potamanthus* clade keeps most original features but penes become complicated and lost color, restricted in the Palearctic realm. The Nearctic genus *Anthopotamus* McCafferty et Bae (1990) is close to the genus *Potamanthus* but their nymphal mandibular tusks are enlarged [22,23]. In Eastern Asia and Oriental realm, where the family Potamanthidae originated, the third clade *Rhoenanthus*+*Potamanthodes* retained the original color on body, with uncomplicated penes but their tusks and forelegs are elongated, spinous row of forefemora appeared, the R_1_ vein of hindwings are curved at base and MP forked asymmetrically. In the latter lineage, the *R. sapa* is an intermediate or linking stage between *Rhoenanthus* and *Potamanthodes*.

McCafferty et Bae (1992) and Mayorga et al. (2024) found some potamanthid nymphs can use their mandibular tusks and forelegs to burrow and fight [15,16], which was not observed in this study of *R. sapa*. This result might be because that we did not rear nymphs in sand but with pebbles and leaves. However, the feeding behavior of *R. sapa* was observed. Besides filtering, the forelegs of *R. sapa* have more functions than *Anthopotamus verticis* [22], like collecting food and moving pebbles.

Vasanth et al. (2024) reported on the *R. sapa* presenting in India [18]. Judging from their pictures, the Indian nymphs have shorter mandibular tusks but bigger tubercles on tusks. Compared to the nymphs in this study, the Indian specimens may not be the real *R. sapa* but a new species. This issue can be tested by its adults and barcoding in the future.

## Figures and Tables

**Table 1:** GenBank ID of *Rhoenanthus* sequences used in this study.

Species	GenBank Accession Number	Provider	Species Origin
*R. sapa*	PZ786720	This study	China
*R.* sp.(1)	HQ581589	Direct submission	Thailand
*R.* sp.(2)	HQ581588	Direct submission	Thailand
*R. coreanus* (1)	MH823267	Direct submission	South Korea
*R. coreanus* (2)	MH823266	Direct submission	South Korea
*R. coreanus* (3)	MH823265	Direct submission	South Korea

**Table 2:** K2P genetic distance among six *Rhoenanthus COI* sequences (%).

Species	*R. sapa*	*R.* sp. (1)	*R.* sp. (2)	*R. coreanus* (1)	*R. coreanus* (2)
*R.* sp. (1)	24.8				
*R.* sp. (2)	24.7	0.3			
*R. coreanus* (1)	26.4	19.9	19.6		
*R. coreanus* (2)	24.5	18.8	18.5	3.2	
*R. coreanus* (3)	23.4	18.3	18.0	3.8	0.6

## Data Availability

All morphological data are presented in this paper, and *COI* gene sequences obtained in this study have been uploaded to the GenBank. The original contributions presented in this study are included in the article. Further inquiries can be directed to the corresponding author.
